# Exploring ethical practice in NGOS on mental health research in Malawi

**DOI:** 10.1371/journal.pgph.0003001

**Published:** 2024-04-11

**Authors:** Action Amos, Cristobal Guerra, Corinne Reid, Edgardo Toro, Clara Calia

**Affiliations:** 1 School of Health in Social Sciences, University of Edinburgh, Edinburgh, United Kingdom; 2 Epilepsy Movers, Blantyre, Malawi; 3 Pan African Network for persons with Psychosocial Disabilities, Blantyre, Malawi; 4 Escuela de Psicología, Facultad de Ciencias Sociales y Comunicaciones, Universidad Santo Tomás, Santiago, Chile; 5 Global Health Academy, University of Edinburgh, Edinburgh, United Kingdom; UNAM: Universidad Nacional Autonoma de Mexico, MEXICO

## Abstract

In recent years, an increasing trend in mental health research has been to collaborate with non-governmental organizations [NGOs] and their constituents. However, ethical difficulties can arise as a result of such partnerships. Understanding the ethics-related practices of NGOs engaged in mental health research is therefore critical. This study addressed these questions in a Malawian context. The goal of this study was to investigate NGO’s ethical practices in relation to mental health research by identifying characteristics that influence ethical practices and investigating staff conceptualization of ethics and mental health. Twenty individuals who work for different local NGOs took part in one-on-one interviews or a workshop about their engagement in diverse research initiatives. They pinpointed the areas that needed improvement, as well as the challenges and chances to create partnerships and increase research capability. The diversity in conceptualizing mental health was a key influence on research practices, with heterogeneity in definitions reflected in the use of cultural, spiritual, behavioural, or medical terms. Notably, there was also a greater emphasis on procedural ethics than ethics-in-practice. Collaboration dynamics and limited staffing capacity were cited as major ethical practice considerations. Each of these elements have an impact on NGOs’ ethical behaviour when conducting mental health research. Participants in the study saw engagement with notions of both ethics and mental health as lacking or rudimentary in their institutions and felt that they needed to be improved through capacity building and stronger research involvement.

## 1. Introduction

While international non-governmental organizations [NGOs] such as the World Health Organization, Christian Aid [1], and Tearfund [2], among others, have clear procedural research ethics rules and criteria, there is a scarcity of literature on practical ethical research procedures for most NGOs. Existing ethics rules are the realm of universities and research ethics committees, and their applicability in NGOs research engagement has not been considered in any systematic way. As a result, this study contributes to our understanding of ethical research practices, with a focus on small NGOs engaging in mental health service provision and mental health research in Malawi.

### 1.1 Ethics in research

Procedural ethics and ethics-in-practice according to Mcevoy [3] refer to the difference that occurs between formal ethical intentions and the practicality on the ground due to the unforeseeble dilemmas that arise during research implementation.

Procedural ethics refers to the formality of governing research and the processes that lead to approval of a research proposal. Procedural ethics often reference established research guidelines [4] which promote key principles of justice, respect and beneficence as per the Belmont report [4]. Procedural ethics are mainly mandated by research review boards in universities, research and medical institutions where review processes are mandatory for all studies involving humans participants. Procedural guidelines provide a regulating framework before research begins, however ethical decision making in research is not static [5], and there is need to also consider ethics *in practice* [6]. The current study will investigate how research ethics are understood, and practised, by NGOs.

Patel [7] highlighted that there is no value addition for society or participants if the research just produces knowledge that is not applied to the advantage of beneficiaries. The key procedural ethical question raised is “who owns the research or who is the research for? [8]. Roth [9] reflected on ethical dilemmas that this brings arguing that not all collaborations are based on fairness and equality. Roth highlighted key questions about the worthiness of the study; end beneficiary of research; unforseen risks; defining of roles and responsibilities; and accountability. Since local researchers and researchers from other countries have different ideas about mental health, there can be "colonialism in research" that affects the ethical principles prioritised.

Tension also often arises between procedural ethics on the one hand and ethics-in-practice on the other [10]. As Tolich [11] stated, ethics committees and researchers cannot forecast the types of ethical difficulties that will arise in the field; all they can say is that they will happen. Ethics-in-practice addresses concerns of field conduct and how research affects all parties involved [12]. A different classification has been suggested by Kelly [13], who proposes four categorizations to ethical thinking: procedural ethics [approval processes], situational ethics [the research context], ethical relationships [dynamics between the researcher and participants] and ethical issues in exiting the study [completion and disseminating findings].

Some additional factors might also influence ethics in practice of NGOs researchers such as lack of knowledge and awareness of proper ethical practices, especially for conducting research in vulnerable populations, thereby increasing the risk of exploitation [14, 15]. Non availability of resources in low and middle-income countries [LMICs] coupled with lack of institutional research capacity and systems contribute to a risk of ethical dilemmas arising and remaining unaddressed among partners. Other ethical challenges are caused by differing perspectives of the researcher and research participants. If views are not reconciled during the designing of the study this can be the source of conflict during research implementation [15]. One of the gaps that affect research comprehension of the subject is that most NGOs do not prioritize research as one of their main focuses [16]. Therefore, the goal of this study was to investigate NGO’s ethical practices in mental health research by investigating staff conceptualization of ethics and mental health and identifying characteristics that influence ethical practices.

### 1.2 Conceptualisation of mental health and mental ill-health as an ethical issue

Conceptualization of the study focus can greatly contribute to ethical dilemmas that might develop at any moment during the research process, including during the phases of data collection, analysis, and reporting [16]. Mental health is a condition of mental wellness that enables people to manage life’s stressors, develop their potential, study and work effectively, and give back to their communities. It is a crucial element of health and well-being that supports both our individual and group capacity to decide, form connections, and influence the world we live in. A core human right is access to mental health. Additionally, it is essential for socioeconomic, communal, and personal development. The absence of mental diseases is only one aspect of mental wellness. It has variable degrees of difficulty and suffering, is experienced differently by each individual, and may have very different social and therapeutic implications. It exists on a complex continuum. Mental health conditions include psychosocial impairments, mental illnesses, and other mental states linked to high levels of suffering, functional limitations, or risk of self-harm. Although this is not always or necessarily the case, people with mental health disorders are more likely to have lower levels of mental well-being [17].

As noted in the literature there are diverse understandings of mental health and mental ill-health that cut across communities and society at large in Africa. Poor mental health might variably be defined as illness, disorder, challenge, or disability in different contexts [16]. These differences apply also to NGO practitioners as mostly they are influenced by factors such as background, culture, history, scientific understanding, and to an extent their mental health literacy among other factors.

According to the predominant medical model mental disorders are the result of physiological conditions [17]. Psychiatrists follow the medical paradigm, addressing mental illnesses as physical ailments that can be diagnosed by a physician and are commonly treated with medicine. Psychologists are more likely to take a biopsychosocial approach to mental ill-health, considering individual temperament, interpersonal and contextual factors in understanding mental health presentations ‐ according to WHO [17,], positive mental health is defined as a condition of well-being in which a person recognizes his or her own potential, is able to cope with everyday stressors, works productively, and contributes to his or her community. Despite its contributions to the study and treatment of mental illness, proponents of the strengths-based perspective and empowerment approaches to mental health practice [18] have criticized the medical model of treatment because it traditionally relies on a largely unidimensional view of mental ill-health, the reliance on expensive medication for treatment, and the assumption of a passive patient who follows orders from health personnel.

There are a variety of alternate mental health cultural notions that are linked to community values, ethics, and behaviours in many African cultures. Personhood is cultivated in Africa through relationships [19]. These connections might be obvious or unseen, and each one can have an impact on a person’s identity. Relationships can take many forms, including spiritual [God, ancestors, and spirits], socially linked [family, clan, and community], and self [a person’s experiences]. This sense of personhood shapes how mental health is conceived in various contexts, including research. According to Kometsi [19], some mental illnesses are said to be caused by supernatural forces or bewitchment, depending on the type of mental illness. Mental health is also seen as a social construct that involves multiple perspectives. The variability can be seen in the nature of the mental illness, its causes, how it manifests and how services are designed to offer treatment.

Research has also shown that stigma driven by culture in Africa has significant secondary influence on ethical practice in research [16]. Goffman, [20] introduced the term courtesy stigma to describe the phenomena of stigma by association, which means that stigma toward a devalued individual generally extends to others who are affiliated with that person, such as family members and professionals, including researchers. Hiflinger [21] supported the notion of stigma by association referring to other categories of mental health professionals indicating that they are undervalued in their respective psychological and medical communities compared to specialists in other subjects.

The conceptualization of mental health and ill-health therefore, can significantly contribute to dilemmas that can arise at any point during the research process, including study design, selection of participants, measures used, data collection processes, analysis, and reporting. The diversity of impacts calls for tailored interventions. In cases when there is no precedent, research itself can assist in creating that precedent for understanding mental health and in ethically responding to research challenges in this field.

### 1.3 The current study

Over 12 non-governmental organizations [NGOs] and four individual support consultants provide mental health services in Malawi. There is no accurate count of the number of persons who access services. There were no indications regarding mental health in the Malawi Population Census of 2018, therefore the total number of people receiving assistance from these organizations is unknown. In Malawi, there is little documented history of collaborative mental health research with NGOs. Gooding [22], highlight that there has been a drive for good collaboration with NGOs beginning in the 1990s’ and early 2000s’, based on the value to global health research. However, conducting culturally sensitive research can be a challenge due to limited capacity, ethical problems and differing cultural perspectives among NGOs [2]. In sum, ethical issues in mental health research collaborations and NGOs research capacity is not well investigated.

The current study focused on ethics in research practices among mental health research NGOs in Malawi throughout the research journey [1]. This study also interrogated understandings of mental health among NGOs and explore how those conceptualizations affect ethical practice in research.

## 2. Methods

### 2.1 Research design

This study used a qualitative design [workshops and indepth interviews] to examine the experiences of NGOs practioners in mental health research.

### 2.2 Research setting and participants

This paper reports interviews with Malawian NGOs that were part of a larger study that focused on NGOs in Africa. Participants were identified through the NGO Board which is the main organisation that regulates the operations of NGOs in Malawi. Fifteen NGO personnel and five key informants in this study were purposively selected [see Table 1 below]. The NGO participants interest was due to their work as researchers who have worked mainly on NGO–academia collaboration as research coordinators, supervisors and managers. Additionally, the participants highlighted that they contribrition in this study would contribute to meaningful engagement and sustainable evidence generation and dissemination on mental health research. Inclusion criteria included that participants are members of the NGO community engaged in mental health research and have at least one year working experience. Participation was voluntary and no compulsion, either explicit or implicit, was used.

**Table 1:** Research participants and their professional roles.

Participant Id	Gender	Individual Expertise	Research Role	Organizational Focus
**1**	**Female**	Mental Health Researcher	Coordinator	Mental Health Counselling
**2**	**Male**	Researcher/Advocate	Officer	Mental Health Advocacy
**3**	**Female**	Health Researcher	Manager	Health Research
**4**	**Female**	Counsellor	Manager	Psychosocial Support
**5**	**Male**	Advocate	Supervisor	Substance Abuse Advocacy
**6**	**Female**	Clinician/Researcher	Coordinator	Mental Health Clinical Service
**7**	**Female**	Psychologist	Coordinator	HIV & Mental Health Service Provision
**8**	**Female**	Health Researcher	Researcher	Research
**9**	**Male**	Researcher	Researcher	Humanitarian
**10**	**Female**	Lawyer	Coordinator	Mental Health Litigation centre
**11**	**Female**	Lecturer in Social Science	Lecturer	Health Service
**12**	**Female**	Trainee Psychologist	Research Assistant	Psychosocial Support
**13**	**Male**	Advocate	Advocate	Substance Abuse Advocacy
**14**	**Female**	Clinician	Researcher	Mental Health Clinical Service
**15**	**Female**	Health Researcher	Coordinator	HIV & Mental Health Service Provision

### 2.3 Materials and methods

This study has received favourable opinion following review by the Edinburgh University, School of Health in Social Science Research Ethics Committee and The National Commission of Science and Technology Research in Malawi.

#### Consent

Potential participants were given a participant information sheet indicating that the research entailed face-to-face interviews with the researcher, and group workshops. The goal of the study was presented and they also received information about their rights as participants, as well as what to expect from the workshop discussions and interview process. Before any data was collected, a consent form was provided and all participants provided verbal and/or written permission. Interviews were conducted primarily in English, with only a little Chichewa mixed. The first author is a native Malawian who speaks Chichewa and is conversant with linguistic code swapping.

#### Research participants

Pre-workshop interviews and then workshop discussions were employed to obtain data for the study. A conversation guide was designed by the researcher to help draw out participants and encourage them to share their experiences.

Some key questions are listed in Table 2 below.

**Table 2:** Conversation guide.

Question#	Question to
1	How would you describe your organization’s engagement with mental health research [producer/collaborator/user/beneficiary, other]?
2	What are some of the ethical research concerns that you have as an organization?
3	Do you have research policy/guidelines in your organization?

A workshop was attended by fifteen persons in Malawi. Malawian participants are included in Table 2 below.

#### Interviews

In order to effectively respond to research questions, five expert interviews with employees from NGOs involved in mental health research were conducted using both online and face-to-face methods. Three women and two men with a bachelor’s degree or above were interviewed. Two sets of interviews [one before and one after the workshop/focus group] were conducted, each lasting around forty minutes. The researcher did not take an expert position over the subjects, enabling them to freely express their feelings and ideas. Key questions are listed Table 3 below.

**Table 3:** Interview guide.

1. In a mental health research collaboration how is the research agenda generated between the partners?
2. What do you think ethically sound research should be like?
3. Do you have any good practice examples?
4. Do you feel current approaches to mental health research resonate with how mental health issues are understood in your context?
5. Do you believe that it is important to include individuals with lived experience in mental health research? If so, how can it be undertaken ethically?

### 2.4 Data analysis

All interviews were transcribed verbatim and, where necessary, translated into English then back translated into the local language by the first author to ensure accuracy and consistency. Data were analysed through thematic analyses using NVIVO qualitative data analysis software. Leedy and Ormrod’s [23] recommended five stages process of thematic analysis were used. The data were classified to highlight the participants’ understanding of research ethics, conceptualisation, capacity and collaboration with other non-NGO actors as broad thematic areas.

## 3. Results

### 3.1 Conceptualization of ethics among mental health NGOs

Our study highlighted that mental health NGOs prioritised different elements of research ethics. Consent, ethical approval compliance, inclusiveness, appropriate study design non-maleficence, and honouring rights were the primary concerns of respondents when describing research ethics. Several respondents mentioned ethics-in-practice and were aware that dilemnas could arise during implementation. However, eight workshop participants commonly referred to *“Standards and principles governing the research established by the ethics committee*,*” “Ethical approvals* …*”*, *and “Consent and Non-Maleficence”* as defining research ethics. This was reflected in both the key informant interviews and group discussions. Despite diversity in description, many of the responses pointed more to procedural ethics than ethics-in-practice, specifically:

#### Full compliance

Six participants [three from the workshops and 3 key informants] indicated that research ethics is characterized by adherence to research procedures established by ethics committees.

"As far as I can tell, it alludes to the national ethics approval committee’s requirement that study protocols be followed… …." [Focus Group Discussion].

#### Inclusiveness

Two workshop participants also highlighted principles of inclusiveness in all research processes including the selection of participants. Respondents highlighted that this is a key aspect because translational research is mainly for the beneficiaries.

"… .… …When you include beneficiaries in all research stages, it demonstrates how ethical the study is."

#### Consent and non-maleficence

Three key informants alluded to respect, inclusion, justice, and fairness as core principles of research ethics that defines research ethics. This was corroborated by two other workshop participants. A principle of freedom of choice and informed consent should be evident in an ethical study. Others interpreted research ethics as the

“………practice of not causing harm to others” [Key Informant].

Evidence of ethical research was defined as being when participants are not endangered as a result of their involvement in the study.

In the workshops some [not quantified] defined research ethics as **Respecting Rights:** It was stated unequivocally that research must ensure that participants and recipients are aware of the study’s goals. Participants and beneficiaries must be informed about their rights as participants, and ethical research must be protected.

In sum, the many different descriptions of research ethics by NGOs are dominated by consideration of procedural ethics [11]. Current debates mostly concern institutional control of procedural ethics.

### 3.2 Conceptualization of mental health among mental health NGOs

The diversified understanding of research ethics is compounded by another diverse notion -what constitutes mental health? Respondents in this study had different conceptualizations of mental health, some focused on cultural and spiritual understanding, others on medical or biological aspects whilst some on absence of psychopathological symptoms.

#### A cause-related understanding

Nine NGO participants stated that mental health is influenced by witchcraft or demonic possession, both of which necessitate spiritual cleansing. This was also stated to be the community’s understanding. They pointed to the fact that this position is most clearly evident when clients are facing a mental health challenge and the first port of call-in seeking help, is the witchdoctor. Mental ill-health was described as; *“punishment from the creator”*, *“a curse that comes your way by chance”*, *“generational evil deeds”*. All 15 participants agreed that this is well known and accepted in the Malawi context. Two respondents in the workshops elaborated that mental illness due to evil spirits was due to;

- Punishment from God
- Evil attacks to disturb someone’s life or intentionally cause them to fail in life [work, marriage or business], bad luck or a curse
- Payment for parents’ evilness

This finding is consistent with the findings from other studies in African countries. Kometsi [19] verifies that mental health in Africa is viewed in terms of its causal form, which is supernatural in character and necessitates spiritual intervention from God or ancestors. Kometsi further emphasises that mental health and mental ill-health is socially connected and has consequences for the family or those who are involved with it.

#### An understanding of behaviour

Behaviour was identified by six workshop participants as a key sign of mental instability that defines mental health. Many respondents revealed that mental health is understood through behavioural signs and symptoms that are judged to be not normal. Participants mentioned that they would know that one is not mentally stable by their speaking and doing abnormal things such as getting angry or acting violence towards people, as *"aggressiveness*,*" "senseless speaking*,*" "eating from bins*,*"* and *"not meeting an acceptable degree of normalcy*.*"* The common belief was that if a person did not engage in appropriate behaviour, they could be labelled as mentally unstable. It was also stated that the source of behavioural issues was caused by witchcraft or demons, as stated in the scriptural or cultural interpretation.

#### Medical comprehension

All participants agreed mental health is the absence of psychopathology, favouring medical-model approaches. Several participants in both key interviews and focus group discussion revealed that they understood mental health to be an illness caused by biological, neurological, inheritance and accidents [17]. Substance abuse was cited as the main cause.

Other studies have also related the absence of psychopathological symptoms to the conceptions of well-being and happiness. Kometsi [19] argued against the medical paradigm, claiming that it fosters passivity in patients.

In sum, there is no unidimensional definition of mental health among NGO workers or amongst the client groups with whom they work. The conceptualization of mental health requires contextual reflections. Additionally, participants in the Malawi workshop acknowledged that their attitudes and actions as researchers and as institutions are influenced by the conceptualization of mental health mainly as an illness, and that mental health research is likely to be stigmatized. Participants indicated that they were hesitant to disclose that they, or members of their family, had been diagnosed with mental disorders. The majority of respondents said they would not disclose this fact to their peers, and some not even discuss it with their spouses or partners, owing to the risk of ostracism and labelling if such revelations were made. They felt that stigma driven by cultural and spiritual beliefs has a significant contribution as disabling factors for them engaging in research. Stigma also limits the timely seeking of support by clients and may worsen the condition further.

This research gives qualitative insights on the link between conceptualisation of mental health and ethical practice among NGOs practitioners. This is, as far as we know, Africa’s first study linking mental health conceptualisation and ethical research conduct in an NGO environment. As discussed, the empirical findings from this study were consistent with earlier research [15, 19] on mental health conceptualization. Mental health, according to Njenga [15] can be regarded as a cultural, social, or medical notion, with terms like mental disease, mental disorder, mental challenge, and mental impairment being used in different settings [15, 19, 24]. Respondents acknowledged that there was no uniformity and that they were influenced by their cultural or professional backgrounds and by the training received in Malawi.

### 3.3 Other factors influencing mental health NGOs ethical practice in research

In addition to examining how understanding of ethics and of mental health influence researchers in NGOs the study also uncovered other significant elements that impede or support ethical practice among of research work in non-governmental organizations. This section considers two significant barriers: collaboration and NGOs capacity.

#### Collaboration

According to the findings, some local NGOs conducting mental health research are concerned about ethical issues such as unequal collaboration and study agenda setting. Key considerations such as *"Power Balance*,*" "Ethical Engagement*,*" "Ownership*,*" and "Agenda Setting"* were mentioned by participants as crucial influencing factors in ethical practice.

**Fig 1:**
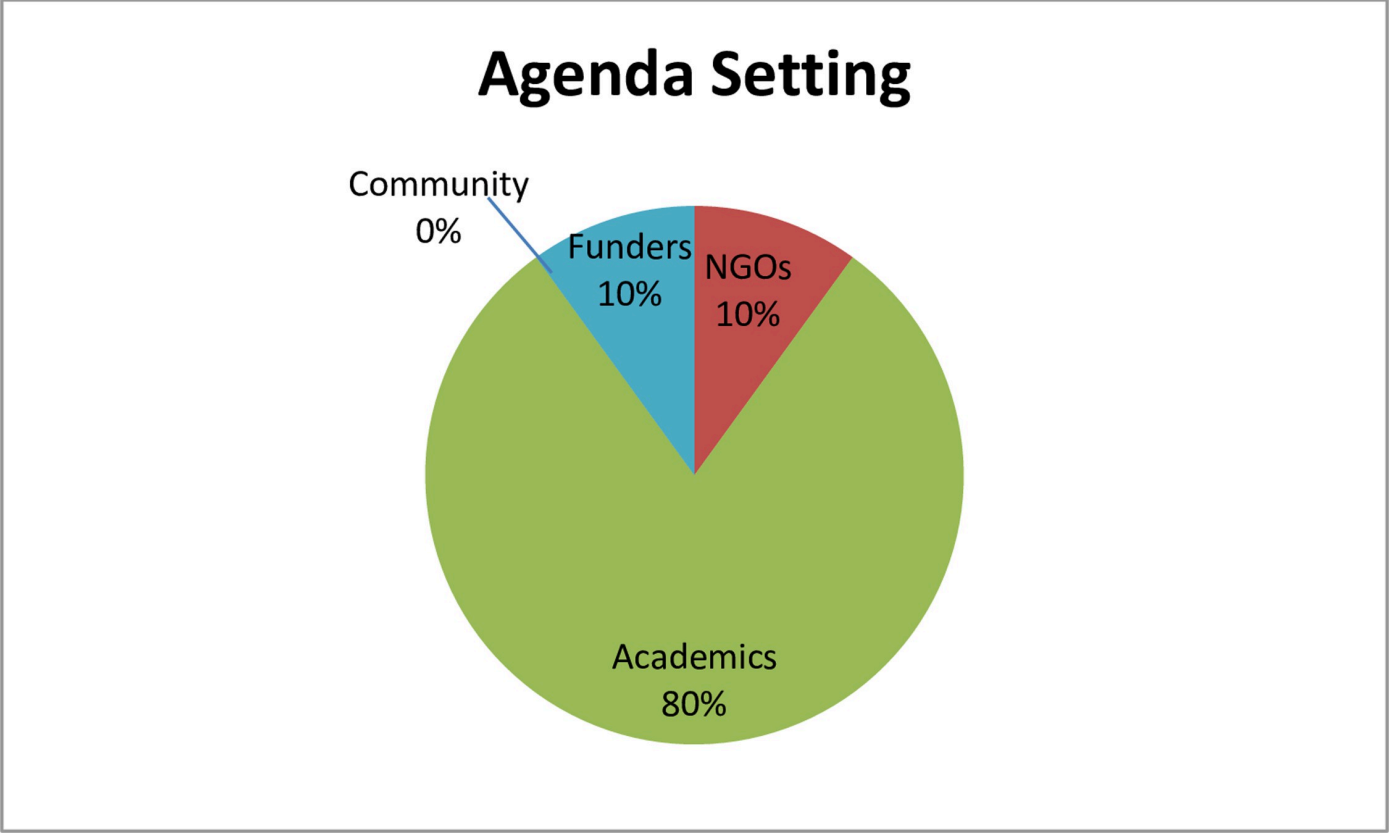
Response from participants on who sets the mental health research agenda from the perspective of NGO practitioners (Workshop, 2021). Eighty percent of the participants said it was academia, ten percent said it was the donors, and the final ten percent said it was the NGOs themselves.

Participants had a diverse clinical and research background [see Table 1] and they were asked about their experience in mental health research collaboration. The participants who are mainly researchers working in an NGO setting related their experience on agenda they had worked on. Most of them admitted that they had collaborated mostly with academics who usually defined the agenda [Fig 1]. Another important theme that emerged was the ability to do research. The NGO participants emphasized that the priorities of funding bodies largely determine what issues and conditions are studied, that agenda-setting is a significant challenge in the governance of health research funding.

Twenty individuals who work for different local NGOs took part in one-on-one interviews or a workshop about their engagement in diverse research initiatives. They pinpointed the areas that needed improvement, as well as the challenges and chances to create partnerships and increase research capability [25, 26].

#### Research capacity

This study reflected a diverse mix of expertise in research capability amongst the NGO’s represented however the capacity of the NGOs’ mental health researchers was described as weak: *“I have high levels of research illiteracy…”*, *“we lack capacity”* and that *“we limited capacity”*. The lack of dedicated people devoted to research was also linked to the NGO capacity issue. According to Fransman [2], research partners’ appreciation of ethical practices makes a difference. It is referred to as institutional research capability, and it can either create or shred research sustainability.

Another contributor to the capacity gap, according to participants, is the lack of organizational research policies or procedures. This is consistent with another study conducted in which respondents identified limited capability along with the lack of organizational research systems as an ethical issue. This was noted to be mainly due to the fact that some NGOs focus would have started without a research focus. Power dynamics’ impact on the development of capacity; the need for deeper ties between research, and practice; and the need of a systems approach to help both capacity and skill. As highlighted by participants capacity building was not included in the alliances and networks that the NGOs collaborated with. Research capacity would have to be built as an ‘add-on’.

## 4. Discussion

### 4.1 Implications for ethical practice

It may be concluded that in most NGO’s in our sample, procedural ethics take precedence over the absence and consideration of ethics-in-practice. Because they largely rely on permissions or procedural ethics, this lack of awareness and ongoing governance may result in a gap in ethics in practice, i.e., NGOs researchers may rely on institutional approvals and may not be well prepared to deal with any emergent concerns in the field. This is especially dangerous in research involving complex conditions or vulnerable individuals, as strict adherence to an established procedure in the face of emergent and shifting needs can lead to manipulation or harm of communities or participants [25].

The above findings on NGO researchers’ interpretations of mental health and ethics have significant implications for mental health research and ethics in practice:

#### Inclusiveness

The majority of respondents agreed that in order to take a comprehensive approach to research, inclusiveness of the diverse client groups with whom NGOs work and the diverse mental health practitioner groups who work from different models should be prioritised, to ensure research is ethical and findings are valid. This study considered how this has an impact on conducting ethical mental health research.

#### Intersection of concepts

Participants suggested that mental health researchers evaluate the conceptualisation of mental health in the place hosting the study, as well as people’s or NGOs’ understandings of mental health. As corroborated by the participants, each of the three understandings interacts with each others, and there is an overall overlap of all three. This factor has significant consequences for researchers because they will need to understand all three concepts in order to design and perform a successful study. As discussed suggesting to that integrating western concepts of psychosocial recovery with non-western understandings would be one of the most difficult problems that requires further research

#### Researchers approach

This creates an ethical dilemma in the field of mental health because NGO researchers should consider all points of view when defining mental health but the current study suggests this is not typical. Views on mental health may differ amongst researchers, between NGO’s and between studies. Attitudes toward persons with mental problems will differ at all levels, such as individual, family, culture, and tribal or nation view. The current study has confirmed Njenga’s [15] finding that cultural and religious beliefs dominate mental health conceptualization in Africa.

#### Passiveness of NGOs and participants

Representatives from non-governmental organizations [NGOs] raised the issue of ownership and sustainability of research finding and application. Because NGOs represent communities, study participants represented by these NGOs can be considered passive research participants. Their understanding of mental health must be explored and understood if ethical standards are to be met. To avoid tokenizing the opinions of local NGOs, co-design, or minimally, ethical alterations to a research design and intervention must be reflective of their input.

#### Stigma

Participants in this study were able to recognize how stigma can affect ethical research practices in mental health. However, many failed to own their own views, which may have influenced stigmatising mental health problems. This is significant since the literature suggests that understanding, identifying, and countering the influence of stigma between a researcher and participants requires awareness of one’s own ideas or prejudices. As discussed stigma by association manifested itself at three levels: intra-personal, [i.e. personal and perceived stigma relating to oneself], inter-personal [when referring to others] and wide [when referring to attitudes and beliefs, emotional reactions, and social distance]. As observed in this study, researchers may not want to associate with those who are afflicted since their own positions may be devalued. This is supported by Goffman [21] who argues that stigma is caused by variables that are understood and interpreted at the individual/psychological, social/community, and societal levels.

In sum, NGO researchers participation in mental health research as collaborators, has significant ethical considerations. The consequences revolve around the research’s usefulness, ownership, and long-term viability. The research will not provide advantages if there is no shared ownership of the knowledge generated within the context of NGOs, regardless of how minimal the risk is as stated in procedural ethics. What is created may be kept in academic files rather than being translated or disseminated to beneficiaries or other custodians such as policymakers. As noted in this study there is need to consider meaningful collaboration where all those involved in research are equal contributors from agenda setting to dissemination. This was emphasised in the development of the toolkit the study by Reid [5] where it is recommend tracking ethical challenges and ethical conduct throughout the research journey. In relation to this study this would be to ensure those NGO researchers and their collaborators are meaningful engaged throughout the research cycle. the toolkit has been developed to support global complex research and as a consequences collaboration between academia and NGO. However, while it received much feedback from global researchers working in academia, we wanted to ask NGOs workers’ opinion. In LMICs usually academics collaborate with NGOs, but access to ethical reflections is usually unbalanced. This is reflected well in the associated toolkit [5] which is designed to support researchers working in complex global contexts to promote higher ethical standards in research, particularly in mental health research. It facilitates ongoing monitoring of the negative consequences of research participation, the interchange of effective methodologies, the application of ethical principles in practice, and the development of new ideas to find solutions. It suggests that researchers should consider ethical challenges in terms of the following factors: 4ps ‐ place [location], people, precedents, and principles. These factors should be mapped at each of 12 key stages [research culture, question formulation, team development, partnership development, application for ethical approval, data collecting, analysis, witing up, knowledge exchange, dissemination, translation to practice, future proofing] of the research journey both prospectively and retrospectively to evaluate progress [5, 27]. This consideration of the inclusive research journey allows NGOs researchers not to be excluded from the onset. Gooding [22] adds that collaborators should think about factors such as competency, capacity building, and respect for partners when deciding who to work with in research.

NGO capability is required to fully engage in mental health research. If capacity is inadequate, ethical gaps may develop. Different elements and levels of capacity are interrelated in mental health research, including connections between factors such as knowledge, skills, and leadership, as well as between individual, organizational, and environmental levels [27]. Kaplan [26] further stated that, in order to ensure that a study is ethical, external interactions should be considered in terms of collaborator capability and power dynamics between partners. Participants also mentioned that if NGOs participate in research without having the necessary capabilities, their claim to desire to be a part of the research agenda formulation may be called into question. That claim’s credibility will be called into question. That claim’s credibility will be called into question. Lack of capability will also have an impact on the amount to which research is adopted [22], such as through promoting policy or behaviour change. Gooding emphasizes the importance of NGOs in Africa having comprehensive research competence that covers the entire research journey. Respondents mirrored this position by stating that prior to the partnership dialogues; research capacity evaluations should be conducted to evaluate organizational strengths and shortcomings [28, 29].

## 5. Conclusion

The goal of this study was to look into the ethical practices of non-governmental organizations in mental health research. This study was undertaken from a developmental standpoint by the first researcher [who previously worked for an NGO and collaborated in research projects on mental health with international institutions]. The study’s aim was to fill a gap in the literature by giving substance and depth to ethical practice through addressing elements that impact or hamper NGOs’ ethical behaviour in mental health research.

Participants in this study provided us with a wealth of information regarding how NGOs handle mental health research in settings, as well as what they think about mental health and how they approach it. The focus of the NGOs’ mental health participants in this study was procedural ethics. It became clear that their shaky definition and inadequate understanding of ethics-in-practice is of concern to them and pervades the mental health NGO sector in Malawi.

On the knowledge about mental health conceptualization, participants contributed a variety of informative insights. Perspectives on mental health and practice ethics were far from exhaustive; it is based on the subjective findings of this qualitative study and is merely designed to synthesize the themes that arose from the data.

Despite the ethical gaps that exist among NGOs involved in research and the implications that this study has identified, there are numerous opportunities for raising awareness and understanding of research ethics, mental health conceptualization, and key barriers to ethical practice among NGOs and other research partners external interactions should be considered in terms of collaborator capability and power dynamics between partners. Participants also mentioned that if NGOs participate in research without having the necessary capabilities, their claim to desire to be a part of the research agenda formulation may be called into question. That claim’s credibility will be called into question.

It is also imperative to conduct ethically acceptable research using transparent approaches when prioritizing agenda items and engaging NGOs. The creation of institutionalized research norms and procedures by non-governmental organizations [NGOs] should be supported. According to the research, many NGOs do not primarily focus on mental health research, and some may lack the ability, technical knowledge, institutional and human expertise, and tools necessary for an organization to conduct successful research and meet future research demands. This demands an investment in training that covers such aspects.

NGOs are currently focused on their work as practitioners, with little capacity to dedicate hours to mental health research. For this reason, the participants in this study refer to collaborations led by the academic world. If NGOs want to carry out more autonomous research, it will require an investment of time and training to acquire the necessary tools and skills, as well as prioritisation of time and opportunity within service design.

There was a consistent view amongst participants that investing in NGO Research Ethics Training, as well as improving mental health research capacity, should be prioritized. There is also the potential to develop an NGO research ethics network in Africa and beyond. A regional ethics network may assist in addressing the donor or academia driven as highlighted in this study distorts ownership and sustainability beyond the research project. Participants pledged to engage with the results of this study as input to assist them to enhance their capacity in research in particular research ethics.

## 6. Limitations

This study offers a preliminary summary of ethical considerations in mental health research in Malawi. The results are not necessarily generalizable because we worked with a small, local [non-representative] sample, however, they do provide local guidance and also indications of where further research might helpfully be directed. This study arose as a starting point in addressing the donor effect in research activity–it is designed to help us better understand the complexities of the service landscape for mental health service provision in Malawi. Beginning with an investigation of a localised contextual perspective is important given the emerging focus on decolonisation of the research agenda–understanding local nuance is the first step in increasing local influence and ownership in mental health research. In turn, this will likely increase the validity and translatability of research findings to local practice.

It is clear that there are differences between the concept of mental health established by different institutions and mental health services, both in Malawi and in other parts of the world. This study has not considered these differences in detail; rather it has focused on investigating the broad landscape of mental health conceptualisation across services. Future studies investigating differential conceptualisations and how they drive or inform service design and delivery would be a valuable addition to the literature. Establishing the efficacy of different treatment approaches and the nature of treatments outcomes would also be helpful, including considering the complementarity of different approaches offered by NGOs and public health services.

This study did not focus on specific mental health conditions or mental health services as this would have limited scope in the research considering that it focused only on Malawi. However more research can be done per region focusing on specific conditions or service types.

Due to its scope, the current study was unable to analyse the level of NGOs’ involvement at each stage of the research process in depth. Further multi-dimensional research on the ethics of collaboration and participation of mental health research stakeholders and beneficiaries is suggested by the researcher.

## Supporting information

S1 File Analysis data files. (DOCX)

S2 File Malawi workshop survey response consolidated. (DOCX)

S3 File Workshop program notes. (DOCX)
