# Correction: Exploring ethical practice in NGOS on mental health research in Malawi

**DOI:** 10.1371/journal.pgph.0004155

**Published:** 2024-12-31

**Authors:** Action Amos, Cristobal Guerra, Corinne Reid, Edgardo Toro, Clara Calia

The affiliation for the fourth author is incorrect. The correct affiliation is not indicated. Edgardo Toro is not affiliated with #4 but with: Pontificia Universidad Católica de Valparaiso, Escuela de Trabajo Social, Chile.
