# Supplementary material for: Exploring ethical practice in NGOS on mental health research in Malawi
Source: PLOS Glob Public Health. 2024 Apr 11;4(4):e0003001. doi: 10.1371/journal.pgph.0003001 (PMC11008845; doi:10.1371/journal.pgph.0003001)
Supplement: S1 File — (DOCX) [file pgph.0003001.s001.docx]

Analysis Data Files

Ethical Challenges

<Files\\Conceptualisation AA> - § 2 references coded [1.86% Coverage]

Reference 1 - 0.88% Coverage

This stigma decrease the likelihood of patients consulting medical services, often favoring the consultation of traditional healers and religious leaders. For this reason, previous research suggests that mental health policies incorporate these different mental health perspectives as well as the different agents validated by the community as mental health promoters

Reference 2 - 0.97% Coverage

Participants reported that they felt both stigmatization and discrimination from by the larger community. To some degree this is a result of a lack of sensitivity by other community members due to a lack of awareness of mental health challenges by the public. The community facilitators expressed the importance of encouraging parents to seek appropriate help in a timely fashion when dealing with children who have mental health issues and disabilities.

<Files\\Ethics in Global Research - WorkshopFollow-up Survey Responses_June 2, 2021_07.25> - § 9 references coded [8.16% Coverage]

Reference 1 - 0.65% Coverage

stress Getting all the people concerned to understand what the research entails. As well as making sure the people

Q

Reference 2 - 0.65% Coverage

raise awareness Come up with ckear cut ethics that take into consideration the cultures and beliefs of each country

Q

Reference 3 - 0.45% Coverage

lack of awareness Cultural , social and religious beliefs should be considered

Q

Workshop Plenary Discussion Understanding Mental Helath

Mental ill-health was described as; “punishment from the creator”, “a curse that comes your way by chance”, “generational evil deeds”. Others related it to behavioural symntoms and other that it was a medical conditions. Others combined the at least two as they indicated it can be a combination. All 15 participants agreed that this is well known and accepted conceptualisation of the large populace in the Malawi.

Reference 4 - 0.70% Coverage

In your context, to what extent are people with lived experience co-creators in mental health research?

Sometimes Seldom

Q1

Reference 5 - 0.67% Coverage

In your context, to what extent is stigma a challenge to mental health research?

Serious challenge Serious challenge

Q1

Reference 6 - 1.31% Coverage

If you consider stigma a minor/moderate/serious challenge, please describe in what ways it is a challenge and how you try to

moderate,Educate Community based awareness and advocacy is required first before embarking on such research.

Q1

Reference 7 - 0.87% Coverage

In your context, to what extent is defining and assessing capacity to consent a challenge to mental health research?

Moderate challenge Minor challenge

Q1

Reference 8 - 1.43% Coverage

If you consider capacity to consent a minor/moderate/serious challenge, please describe in what ways it is a challenge and ho

raise understanding Most of the people are struggling to make a living, as long as they are offered some form of compensation for

Q1

Reference 9 - 1.43% Coverage

In your context, are there any other limits or barriers to the inclusion of people with lived experience in mental health resear

change policies The leaders of the area might also want to profit in this and so might not be willing to have people do research

<Files\\Ethics_in_Global_Research_-Malawi Workshop Follow up Survey> - § 7 references coded [35.42% Coverage]

Reference 1 - 4.95% Coverage

**Individual 1**: As mental health is not a very common subject in Malawi, it is hard to meet a targeted group in this regard.

**Individual 2**: mental health is misunderstood, hence excluded or receive less attention among stakeholders and community participants.

**Individual 3&9&10:** Limited financial and technical support

**Individual 4:** lack of research in Malawi as all the topics and target groups NGOs are working on do not focus on mental health

**Individual 5&8:** the emphasis is put on nutrition and does not focus on the underlying causes of malnutrition which may be related to mental health state of caregivers.

**Individual 6:** the most significant challenge is that issued of mental health are neglected and not given equal interest as others

Reference 2 - 2.05% Coverage

**Q11 In your context, to what extent are people with lived experience co-creators in mental health research? o Often (1) o Sometimes (2) o Seldom (3) o Never (4)**

**Individual 1**: Sometimes

**Individual 2**: sometimes

**Individual 3:** sometimes

**Individual 4:** sometimes

**Individual 5:**  Often

**Individual 6:** Often

**Individual 7**: sometimes

**Individual 8:** sometimes

**Individual 9:** sometimes

**Individual 10:**  Often

**Individual 11:** Often

Reference 3 - 8.55% Coverage

**Q13 If you consider stigma a minor/moderate/serious challenge, please describe in what ways it is a challenge and how you try to overcome it in your context?**

**Individual 1**: Mental Health is rarely discussed in Malawi and a lot of people do not understand it in that sense. A person who is suicidal is not looked at as a person with mental health. Civic education on the matter is very vital

**Individual 2**: it is a serious challenge because it lacks supportive interventions it deserves. We try to create public awareness about it through our networks for civil society organizations and our project supported areas.

**Individual 3:** those suffering hide out and pretend they are normal. We can overcome that by sensitizing people that mental health is like any other disease.

**Individual 4:** people fail to open up on their mental issues because the platforms are insufficient

**Individual 5:** the society tends to judge people with mental health issues, as such those issues are suppressed but exist so much in our midst. Having proper platforms to address these issues would help so much while also protecting the affected people.

**Individual 6:** those with mental health are regarded as weak and not taken as real men. We try to raise awareness of communities on mental health.

**Individual 7:** Those in pain hide away and act normally. By making people aware that mental health is just like any other condition, we can overcome that.

**Individual 8:** Because there aren't enough outlets, many are reluctant to discuss their mental health problems.

**Individual 9:** Mental health problems are concealed yet are very prevalent in our culture because of the stigma attached to them..

**Individual 10:** Males could be struggling with mental illness and would not come for pschosocial support

Reference 4 - 2.83% Coverage

**Q14 In your context, to what extent is defining and assessing capacity to consent a challenge to mental health research? o Not at all a challenge (1) o Minor challenge (2) o Moderate challenge (3) o Serious challenge (4)**

**Individual 1:** Serious Challenge

**Individual 2:** serious challenge

**Individual 3:**  serious challenge

**Individual 4:**  serious challenge

**Individual 5:** moderate challenge

**Individual 6:** moderate challenge

**Individual 7:**  serious challenge

**Individual 8:**  serious challenge

**Individual 9:** moderate challenge

**Individual 10:** moderate challenge

Reference 5 - 7.81% Coverage

**Q15 If you consider capacity to consent a minor/moderate/serious challenge, please describe in what ways it is a challenge and how you try to overcome it?**

**Individual 1**: Not only are people not aware of mental health, even those with mental health are not aware. Therefore, this creates challenges in finding participants.

**Individual 2**: it is a challenge because consequently the mental health challenges would not be

We try to explain in clear terms on the importance of defining and capacity to consent so that mental health research is done in accordance with the research ethics.

**Individual 3:** they are afraid of being stigmatized- we can overcome the problem by teaching mindset change to society

**Individual 4:** lack of awareness on the assessment or defining mental health statuses.

**Individual 5:**  when we go to communities, we often don’t explain in detail the intent, we assume they are always ready and willing to attend to us and answer our questions. So, we have to explain in details for their understanding and allow them to make an informed decision.

**Individual 6:** by letting people know all the criteria involved in mental health counseling

Reference 6 - 6.11% Coverage

**Q17 In your context, does mental health research receive sufficient resources? o Yes (1) o No (2)**

**9** individuals= No

**Q18 If no, please describe what resources are needed and what impact you think this has?**

**Individual 1**: NGOs Financial capacity, personnel, research capacity. This lack of inadequate of the above-mentioned resources slows down the work.

**Individual 2: As NGOs**  there are inadequate human resources and financial resources which create negative impacts in addressing the mental health challenges.

**Individual 3:** financial and technical support needed to ensure that these challenges are addressed.

**Individual 4:**  NGOs tend to ignore it and focus on their issues

**Individual 5:** Capacity and funds to run programs that support mental health, in order to address mental health issues in our communities.

**Individual 6:** need for expertise to train people who deal with mental health issues. “We lack capability, we have limited capacity, and I have significant degrees of research illiteracy.”

**Individual 7-10:** Capacity to be involved in research is limited as this mainly would not be our main focus whe our NGOs were setuo

Reference 7 - 3.12% Coverage

**Q20 If none of these, please explain further. If more than one, how are the priorities different?**

**Individual 1:** The academia, medical profession provides the information through research findings. The government develops policies and approves the implementation. The civil society advocates for the implementation of such policies.

**Individual 4:** government and civil society need to work on policy implementation and the academia needs to conduct more research.

Reference 1 - 27.27% Coverage

Individuals 5, 9,10: discrimination of persons with mental health challenges in LMICs.

Individuals2, 3, 6, 7,8, 11: Persons with mental health challenged or NGOs are not consulted on their research needs since they are defined as not well.

<Files\\NGOPAK> - § 2 references coded [3.03% Coverage]

Reference 1 - 1.26% Coverage

biggest challenges that we have. sometimes it's limited engagement of NGOs that represent people from the scientist now, even though we are available to engage but it's at what point we have been engaged and we are being asked to come on the space.

Reference 2 - 1.77% Coverage

And lastly these concerns to donors and academicians, I am not against them. But for them to be inclusive in research sovereignty I think that lacks. There is a bit of not resentment not accepting it which in a way defeats the actual part of the ethical nature of the research.

<Files\\NGOYON> - § 1 reference coded [2.04% Coverage]

Reference 1 - 2.04% Coverage

how NGOs can develop their own research units because one of the things that is happening is that there is a lot of research that people are doing maybe in the form of Baseline surveys in form of collecting data, and doing some report at the end of the year and again there's also a lot of data that is being collected inform of research about our work and the question is what do those results mean to the organization.

<Files\\REPORT> - § 1 reference coded [3.97% Coverage]

Reference 1 - 3.97% Coverage

Mental Health

Challenge, unable to give consent and lack of mental health awareness

Solution

Raise awareness mental health .

Those were the findings.

At the end everyone was therefore conversant with the ethics in global research issue.

Files\\Ethics_in_Global_Research_-Malawi Workshop Follow up Survey> - § 3 references coded [12.53% Coverage]

Reference 1 - 0.60% Coverage

In your context, does mental health research receive sufficient resources?

No No - Capacity is lacking and more important

Q1

Reference 3 - 0.82% Coverage

In your context, who sets the priorities for mental health research?

Academia and donors

Q2

<

Reference 1 - 1.07% Coverage

**Q9 Do you consider current clinical frameworks for understanding mental health are sufficient for working in your context? o Yes (1) o No (2)**

**9** individuals= No

Reference 2 - 4.66% Coverage

**Q10 If not, please explain**

**Individual 1**: There is inadequate material on mental health

**Individual 2**: mental health agendas are not adequately mainstreamed in the program design and implementation of interventions and are rarely (rarely) talked of or appreciated as a cross cutting issues in the programming that target the commanding.

**Individual 3:** not prioritized

**Individual 4:** because the clinical platforms in Malawi do not exist and not sufficient enough

**Individual 5:** because as it is at the moment, no efforts are being directed to mental health related issues.

**Individual 6:** because mental health issues are regarded as issues for Americans or Europeans and not strong black people

Reference 3 - 6.79% Coverage

**Q21 In your context, do you think that religious, spiritual and other local belief systems have a role in mental health research? o Yes (1) o No (2)**

10 individuals= Yes

**Q22 If yes, how would you describe this role?**

**4 individuals:** stigma is high and suffocates all other efforts on

1. Individual
2. Institutions
3. Society at large

**Individual 1:** These systems are influencers, therefore have a role to play in the provision of the right information to people

**Individual 2:** Realizing and appreciating that mental health challenges affect the congregants and followers of religious and spiritual groupings they have a significant role to play in creating awareness and managing mental health among followers

**Individual 3:** to enable their followers change their mindset and starting regarding mental health as a disease like any other diseases.

**Individual 4:** they influence the mental statuses

**Individual 5:** awareness of the problem to mentors

**Individual 6:** religious and local leaders are trusted by communities where they rule issues, so they can be used to counsel those with mental health issues.

<Files\\NGOYON> - § 1 reference coded [1.18% Coverage]

Reference 1 - 1.18% Coverage

the challenge is that if the data that you give to me cannot be translated in any form then what's it for. So in other words what I'm saying is how can NGOs translate the information that they collect into something meaningful that will inform

<Files\\Workshop Review Themes Docs - § 1 reference coded [1.63% Coverage]

<Files\\REPORT> - § 1 reference coded [27.28% Coverage]

Reference 1 - 27.28% Coverage

People spoke their views in groups and one on one answering how they understood research ethics.

Participant 2: Before research get **consents**, if the participants required are under age it is very important to get consent from their parents. Participants also need to be given all the respect they need regardless of their background and everything concerning both their physical or mind outlook.

Participant 1: the research should benefit the people in their **communities respectively**. We also have to consider if the people are **participating** in the research or not. If there not participating they cannot be ethics.

Participant 3:transparency

This is very important because people have to know what they are involving themselves in. **transparency** is key

Participant 4: accountability

We should and ought to be held accountable for the proceedings of the research.

Workshop Group Discussion Pairs.

1)Standards and principles guiding the research, ie; the **rights of the participants** and the researchers.2) Research process be **beneficial to the communities**, ie; vulnerable groups have to be protected.3) **Ethics guidelines**, Getting approval from the National ethics committee.4) Getting consents from the participants.4) Working in respect of cultural and religious beliefs. 5) Transparency, disclosure of information about the research. 5) Accountability to the community about the proceedings of the research.5) **Non Maleficence**, **do not harm.** 6) **Beneficence,** do good. 7) **consent** and many others were put forward.

"As far as I can tell, it alludes to the national ethics approval committee's requirement that study protocols be followed......." (Focus Group Discussion).

In plenary individual provided feed back

“Standards and principles governing the research established by the ethics committee, this is more to do with ethical committee approvals,” Individual 3

"This is a bit confusing confusing because coming from a background of health and human rights rights ethics are observed when you include beneficiaries in all research stages, It demonstrates how ethical the study is." Individual 5

“As paer my training in clinical practice it is the practice of not causing harm to others when patient are the clinical setting” (Key Informant/Indivdual 6).

<Files\\Ethics_in_Global_Research_-Malawi Workshop Follow up Survey> - § 7 references coded [31.27% Coverage]

Reference 1 - 6.27% Coverage

**Q8 Can you describe what significant opportunities for enhancing ethics in mental health research might exist in your context?**

**Individual 2**: Considering the increase in incidences resulting from mental health problems among adolescent youth and reproductive men and women enhancing ethics in mental health will created significant impact in interventions that address mental health related challenges

**Individual 3:** Linkages that exists with international partners in this field

**Individual 4:** research and then implementing platforms to manage mental health issues. Additionally, government implementing policies on mental health.

**Individual 5:** incorporating a mental health policy into the already existing policies and have more activities that address mental health issues.

**Individual 6:** first thing should be to raise awareness in mental health issue so that players come in to address the issues by first conducting research.

Reference 2 - 1.07% Coverage

**Q9 Do you consider current clinical frameworks for understanding mental health are sufficient for working in your context? o Yes (1) o No (2)**

**6** individuals= No

Reference 3 - 4.66% Coverage

**Q10 If not, please explain**

**Individual 1**: There is inadequate material on mental health

**Individual 2**: mental health agendas are not adequately mainstreamed in the program design and implementation of interventions and are rarely (rarely) talked of or appreciated as a cross cutting issues in the programming that target the commanding.

**Individual 3:** not prioritized

**Individual 4:** because the clinical platforms in Malawi do not exist and not sufficient enough

**Individual 5:** because as it is at the moment, no efforts are being directed to mental health related issues.

**Individual 6:** because mental health issues are regarded as issues for Americans or Europeans and not strong black people

Reference 4 - 2.57% Coverage

**Q12 In your context, to what extent is stigma a challenge to mental health research? o Not at all a challenge (1) o Minor challenge (2) o Moderate challenge (3) o Serious challenge (4)**

**Individual 1**: Serious Challenge

**Individual 2**: serious challenge

**Individual 3:** serious challenge

**Individual 4:** serious challenge

**Individual 5:** serious challenge

**Individual 6:**  serious challenge

Reference 5 - 8.55% Coverage

**Q13 If you consider stigma a minor/moderate/serious challenge, please describe in what ways it is a challenge and how you try to overcome it in your context?**

**Individual 1**: Mental Health is rarely discussed in Malawi and a lot of people do not understand it in that sense. A person who is suicidal is not looked at as a person with mental health. Civic education on the matter is very vital

**Individual 2**: it is a serious challenge because it lacks supportive interventions it deserves. We try to create public awareness about it through our networks for civil society organizations and our project supported areas.

**Individual 3:** those suffering hide out and pretend they are normal. We can overcome that by sensitizing people that mental health is like any other disease.

**Individual 4:** people fail to open up on their mental issues because the platforms are insufficient

**Individual 5:** the society tends to judge people with mental health issues, as such those issues are suppressed but exist so much in our midst. Having proper platforms to address these issues would help so much while also protecting the affected people.

**Individual 6:** those with mental health are regarded as weak and not taken as real men. We try to raise awareness of communities on mental health.

Reference 6 - 4.62% Coverage

**Q16 In your context, are there any other limits or barriers to the inclusion of people with lived experience in mental health research and how do you overcome these barriers?**

**Individual 2**: no there are no any other limits or barriers.

**Individual 3:** there are several barriers; Not considered as a development issue by the government and its partners. Not supported financially.

**Individual 4:** through awareness campaigns

**Individual 5:** yes, stigma. Allocate more attention and support to mental health issues.

**Individual 6:** due of the barriers is cultural values perceive one with mental health as weak. The solution is to sensitize communities that everyone can have mental problems.

Reference 7 - 3.52% Coverage

**Q19 In your context, who sets the priorities for mental health research? ▢Academia (1) ▢Civil Society (2) ▢Government (3) ▢Medical Profession (4) ▢People with Lived Experience (5) ▢None of these (6)**

**Individual 1,2,7,8,9,10 :** Academia and others(donors)

**Individual 3:** Academia, civil society, medical profession, people with lived experience

**Individual 4:** academia, civil society, government, medical profession, people with lived experience

**Individual 5:** government

**Individual 6:** civil society, medical profession, people with lived experience

<Files\\NGOMENT> - § 1 reference coded [0.87% Coverage]

Reference 1 - 0.87% Coverage

NGO not setting agenda .But the academics coming from the other institutions set agenda. We need to be fair everyone needs to be part and not tokenism participation

<Files\\Ethics in Global Research - Africa Workshop Follow-up Survey Responses_June 2, 2021_07.25> - § 1 reference coded [0.65% Coverage]

Reference 1 - 0.65% Coverage

raise awareness Come up with ckear cut ethics that take into consideration the cultures and beliefs of each country

Q

<Files\\MSC Review Themes Doc> - § 8 references coded [20.62% Coverage]

Reference 1 - 3.46% Coverage

Non availability of resources in low and middle-income countries (LMICs) coupled with lack of institutional research capacity and systems contribute to a risk of having ethical dilemnas among partners when conducting research. Other ethical challenges in research are caused by differing perspectives of the researcher and the research participants.

Reference 2 - 1.63% Coverage

the choices for setting the research agenda are limited, as the thematic areas eligible for grants are generally determined by the interests of funder or the researcher (Siraj,2006)

Reference 3 - 1.41% Coverage

NGOs in the LMICs lack capacity to be involved throughout the research journey especially when it comes to packing the results for uptake by the end users.

Reference 4 - 3.00% Coverage

Those without research guides may rely on organisational codes of conduct as guides to support conduct during research (Kanyegere, 2019). These guides could be merely be human resource protocols to assist with security or good behavior during project with emphasis on do no harm to project beneficiairies but not research related.

Reference 5 - 2.72% Coverage

It has been noted that in some of the research there is no societal or participants value addition if the research only generates knowledge that is not translated to the benefit of participants or the beneficiaries. The key ethical question raised is; who owns the research or who is the research for?

Reference 6 - 2.20% Coverage

Another ethical challenge that has been highlighted is that of non-familiarity with research. Communities may construe that the data collection is meant to be part of mitigating initiative to their plight with anticipation of a quick solution.

Reference 7 - 2.27% Coverage

discrimination of persons with mental health challenges in LMICs. Persons with mental health challenged are not consulted on their research needs since they are defined as not well. Substituted decision making therefore used using close member families

Reference 8 - 3.92% Coverage

Users or persons with mental health challenges are further stigmatized by those that are supposed to engage with in research in that their legal capacity is challenged (Siriwardhana, 2015). There is another ethical risk of caused by not contextualizing research to suit populations whose characteristics such as gender, age and sex are different but defined by the same mental challenge or geographical location (Ruiz-Casares, 2014).

<Files\\NGOgangdrc> - § 3 references coded [10.71% Coverage]

Reference 1 - 3.18% Coverage

So we have noticed that when we do research we have noticed that our clients They Come For All the sick sessions because of the reimbursements that they get after they do a session so they come because of that. Then after the research it's now difficult for us to get clients to come and do all the 6 sessions

Reference 2 - 2.88% Coverage

Yes I think one of the challenges that we faced was on sample size. Like before you start doing research before you even go in the field, you are just told that we need a sample size of this much. Then maybe towards the end as you are about to reach your sample size, it then changes

Reference 3 - 4.65% Coverage

so sometimes you just end up having this conflict with participants and some of them they don't understand why they've not been accepted in the study because don't reveal the criteria that we're using to recruit because once you tell them about the criteria they go and tell others then people may come and lie so then to fit in the study so we do not disclose a criteria maybe we'll only disclose cuts of the criteria when someone has not been successful

<Files\\NGOMENT> - § 2 references coded [1.68% Coverage]

Reference 1 - 0.79% Coverage

first ethical consideration is in this research whatever you're researching whose benefit is that . Is it for academic institution's benefit.

Y

Reference 2 - 0.89% Coverage

NGO stopped being paid.But the academics coming from the other institutions get paid. We need to be fair everyone needs to be receiving and not just gratitude.And

<Files\\NGOPAK> - § 4 references coded [10.36% Coverage]

Reference 1 - 3.21% Coverage

It can never really be a level play field mostly because research is a very professional space and often times communities I seen as noisemakers where research is concerned. So yeah, I think there are a lot of challenges first of all I would talk about research literacy to ask as an organization but also for the communities we work with because I can confidently say I've been one of the one of the people who is sitting in these high-level meetings but it never gets easy the science sometimes is too much so the research literacy is quite a big Gap.

Reference 2 - 3.07% Coverage

So, we kind of come in to rubber stamp things, we are not really involved in the beginning of the process. As they are starting the process, they don’t engage us in the middle of the way, it's just, we have these results what do you think. And because most of the times we had not been involved, it's really difficult to engage at that time. So, you just say, well this looks good when actually we really don’t have a clue what they are talking about. So, I think level of engagement and depth of engagement is quiet a big issue.

Reference 3 - 2.32% Coverage

I'll take a slight departure on objectivity and issue of paying out to the respondents. Sometimes I have participated in research where you keep people for two to three hours you don’t provide them with anything because you said if we provide them with something, they’ll twist the information that they should provide. But I am saying as humans are we subjecting them to the right kind of treatment.

Reference 4 - 1.77% Coverage

And lastly these concerns to duty bearers, I am not against them. But for them to assimilate the research findings and take them with maturity and sovereignty I think that lacks. That is a bit of not resentment not accepting it which in a way defeats the actual part of the ethical nature of the research.

<Files\\NGOredcar_1> - § 1 reference coded [3.58% Coverage]

Reference 1 - 3.58% Coverage

one of the ethical considerations is religious beliefs of the communities that we want to engage them in.

Another ethical consideration is that one of cultural aspect.

<Files\\NGOthandimal> - § 2 references coded [4.99% Coverage]

Reference 1 - 1.26% Coverage

I think we've been used more as turtle nest group or organization just so that they're following the principles for production.

Reference 2 - 3.72% Coverage

But then like you said they are side-lined they just used for the moment when they want the information, they do the interview and then the people disappear. Then that’s also not helpful because then they say we want users to be empowered so that they can function in society. So those research policies can have strong components of capacity building. That will also be great.

<Files\\NGOYON> - § 4 references coded [5.32% Coverage]

Reference 1 - 2.04% Coverage

how NGOs can develop their own research units because one of the things that is happening is that there is a lot of research that people are doing maybe in the form of Baseline surveys in form of collecting data, and doing some report at the end of the year and again there's also a lot of data that is being collected inform of research about our work and the question is what do those results mean to the organization.

Reference 2 - 1.18% Coverage

the challenge is that if the data that you give to me cannot be translated in any form then what's it for. So in other words what I'm saying is how can NGOs translate the information that they collect into something meaningful that will inform

Reference 3 - 0.68% Coverage

how do we do match how can NGOs be involved in research that brings out the issues that are then translated into practice to solve problems.

Reference 4 - 1.42% Coverage

And therefore building capacity for local organizations in conducting and interpreting research information would be key for universities and could be Partnerships that would need organizations to be able to design studies maybe in partnership with academic institutions and then the academic

Workshop Group Plenary Discussion

Key Factors affecting ethical practice displayed on charts(summarised)

- *"Power Balance,"*
- *"Ethical Engagement,"*
- *"Ownership,"*
- *"Agenda Setting"*

<Files\\Workshop - § 1 reference coded [1.63% Coverage]

Reference 1 - 1.63% Coverage

the choices for setting the research agenda are limited, as the thematic areas eligible for grants are generally determined by the interests of funder or the researcher

<Files\\NGOthandimal> - § 1 reference coded [0.78% Coverage]

Reference 1 - 0.78% Coverage

I can't say I like it, I don't like it, I don't think there is a Power Balance

<Files\\REPORT> - § 1 reference coded [12.57% Coverage]

Reference 1 - 12.57% Coverage

**Ethical Dilemmas**

Ethical dilemmas were also discussed in groups and the following were their findings.

1. Late approvals

Mitigation: going ahead without approvals

2.Inadequate support

Mitigation

Relocation of the activity.

2.Financial demands

Mitigation: Stick to our budget regardless

3.Acceptance of research findings

Mitigation: stick with our results.

4.To follow set rules and policies

Mitigation: Doing follow ups

5. Collaboration with other partners on how they manage research problems and challenges.

Another group came up with the following

1.Limited time

Mitigation: have a readily available research team.

2. Inadequate funding

Mitigation: have strains of funding.

3.Lack of expertise in research ethics

Train the team in research ethics
