# Supplementary material for: Exploring ethical practice in NGOS on mental health research in Malawi
Source: PLOS Glob Public Health. 2024 Apr 11;4(4):e0003001. doi: 10.1371/journal.pgph.0003001 (PMC11008845; doi:10.1371/journal.pgph.0003001)
Supplement: S2 File — (DOCX) [file pgph.0003001.s002.docx]

**MALAWI WORKSHOP SURVEY RESPONSE CONSOLIDATED.**

**Q1 Consent:** All 12 respondents consented to all 5 options

**Q2 Role in researcher –**All 12 indicated option 2 I am a researcher in an NGO/Civil Society

**Q3 Country –** Malawi for all 12

**Q4 Career Stage –** all we not post PHD and the researcher and participants agreed to skip as reference was mainly Post PHD and all had no PHDs

**Q5 In what discipline or area do you work?**

**Individual 1**: Health research

**Individual 2**: Health (HIV/ Aids) and Human Rights

**Individual 3:** Mental Health and Alcohol and Drugs

**Individual 4:** Public Health and HIV/AIDS Advocacy

**Individual 5:** Health and Nutrition Advocacy

**Individual 6:** Planning, Quality and Learning which is more of an M and E discipline

**Individual 7:**  Public health

**Individual 8:** Health Advocacy

**Individual 9:** Advocacy

**Individual 10:** Advocacy

**Individual 11:** Advocacy

**Individual 12:** Health

**Q6 Do you consider yourself to be working in the area of mental health? o Yes (1) o No (2)**

**11** individuals= yes - all – respondents expect respondent 6

1 Individual= No – respondent 6

**Q7 Can you describe what significant ethical challenges for mental health research exist in your context?**

**Individual 1**: As mental health is not a very common subject in Malawi, it is hard to meet a targeted group in this regard, the research agenda as discussed is not owned by NGOs.

**Individual 2**: mental health is misunderstood, hence excluded or receive less attention among stakeholders and community participants. Additionally capacity of NGOs to carry research adhering or following all stages is an ethical issue

**Individual 3:** Limited financial and technical support (capacity) are the issues that affect ethical practice

**Individual 4:** lack of research in Malawi as all the topics and target groups NGOs are working on do not prioritize mental health. Ownership of research when done is not sustainable

**Individual 5:** the emphasis is put on nutrition and does not focus on the underlying causes of malnutrition which may be related to mental health state of caregivers.

**Individual 6:** the most significant challenges are that issued of mental health are neglected and not given equal interest as others, setting of research agendas are not inclusive.

**Individual 7:** There is a lot of stigma and non-tolerance of persons with mental health challenges and research capacity of NGOs

**Individual 8:** Stigma and discrimination are the biggest barriers among researchers and communities

**Individual 9:** I have partly worked in this area I think issues to do with research capacity of NGOs is a challlenge

**Individual 10:** Engagaiging persons with mental challenges has been the major ethical challenge. We have to use third parties to represent them.

**Individual 11:** The issue of legal capacity has been the major challenge. When working with communities with these challenges how to engage thems is a problem.

**Individual 12:** did not answer

**Q8 Can you describe what significant opportunities for enhancing ethics in mental health research might exist in your context?**

**Individual 1:** Capacity building for Ngos in mental health research and ethics

**Individual 2**: Considering the increase in incidences resulting from mental health problems among adolescent youth and reproductive men and women enhancing ethics in mental health will created significant impact in interventions that address mental health related challenges

**Individual 3:** Linkages that exists with international partners in this field and capacity building of NGOs

**Individual 5:** research and then implementing platforms to manage mental health issues. Additionally, government implementing policies on mental health.

**Individual 7:** incorporating a mental health policy into the already existing policies and have more activities that address mental health issues.

**Individual 8:** first thing should be to raise awareness in mental health issue so that players come in to address the issues by first conducting research.

**Individual 9:** Create a platform for ethics in practice discussion

Respondents 4, 6, 10, 11, 12 did not answer.

**Q9 Do you consider current clinical frameworks for understanding mental health are sufficient for working in your context? o Yes (1) o No (2)**

**7** individuals= No

Respondents 4, 6, 10, 11, 12 did not answer.

**Q10 If not, please explain**

**Individual 1**: There is inadequate material on mental health

**Individual 2**: mental health agendas are not adequately mainstreamed in the program design and implementation of interventions and are rarely (rarely) talked of or appreciated as a cross cutting issues in the programming that target the commanding.

**Individual 3:** not prioritized

**Individual 5:** because the clinical platforms in Malawi do not exist and not sufficient enough

**Individual 7:** because as it is at the moment, no efforts are being directed to mental health related issues.

**Individual 8:** because mental health issues are regarded as issues for Americans or Europeans and not strong black people

Individual 7: **no response**

Respondents 4, 6,9, 10, 11, 12 did not answer.

**Q11 In your context, to what extent are people with lived experience co-creators in mental health research? o Often (1) o Sometimes (2) o Seldom (3) o Never (4)**

**Individual 1**: Sometimes

**Individual 2**: sometimes

**Individual 3:** sometimes

**Individual 4:** sometimes

**Individual 5:**  Often

**Individual 6:** Often

**Individuals 7 – 12** Never

**Q12 In your context, to what extent is stigma a challenge to mental health research? o Not at all a challenge (1) o Minor challenge (2) o Moderate challenge (3) o Serious challenge (4)**

**Individual 1**: Serious Challenge

**Individual 2**: serious challenge

**Individual 3:** serious challenge

**Individual 4:** serious challenge

**Individual 5:** serious challenge

**Individual 6:**  serious challenge

**Individual 7**: Serious Challenge

**Individual 8**: serious challenge

**Individual 9:** serious challenge

**Individual 10:** serious challenge

**Individual 11:** serious challenge

**Individual 12:**  serious challenge

**Q13 If you consider stigma a minor/moderate/serious challenge, please describe in what ways it is a challenge and how you try to overcome it in your context?**

**Individual 1**: Mental Health is rarely discussed in Malawi and a lot of people do not understand it in that sense. A person who is suicidal is not looked at as a person with mental health. Civic education on the matter is very vital

**Individual 2**: it is a serious challenge because it lacks supportive interventions it deserves. We try to create public awareness about it through our networks for civil society organizations and our project supported areas.

**Individual 3:** those suffering hide out and pretend they are normal. We can overcome that by sensitizing people that mental health is like any other disease.

**Individual 5:** people fail to open up on their mental issues because the platforms are insufficient

**Individual 7:** the society tends to judge people with mental health issues, as such those issues are suppressed but exist so much in our midst. Having proper platforms to address these issues would help so much while also protecting the affected people.

**Individual 8:** those with mental health are regarded as weak and not taken as real men. We try to raise awareness of communities on mental health.

**Individual 9:** There is need for holistic approach to ensure there is awareness of mental health research

**Individual 11:** NGOs need to improve capacity of engagement in mental health research and ethics

**Individual 12:** All gatekeeper at community level should be involved

Respondents 4, 6, 10, did not answer.

**Q14 In your context, to what extent is defining and assessing capacity to consent a challenge to mental health research? o Not at all a challenge (1) o Minor challenge (2) o Moderate challenge (3) o Serious challenge (4)**

**Individual 1:** Serious Challenge

**Individual 2:** serious challenge

**Individual 3:**  serious challenge

**Individual 4:**  serious challenge

**Individual 5:** moderate challenge

**Individual 7:** moderate challenge

**Individual 8:** serious challenge

**Individual 9:**  serious challenge

**Individual 10:**  serious challenge

**Individual 11:** moderate challenge

**Individual 12:** moderate challenge

Individual 6 – did not respond

**Q15 If you consider capacity to consent a minor/moderate/serious challenge, please describe in what ways it is a challenge and how you try to overcome it?**

**Individual 1**: Not only are people not aware of mental health, even those with mental health are not aware. Therefore, this creates challenges in finding participants.

**Individual 2**: it is a challenge because consequently the mental health challenges would not be

We try to explain in clear terms on the importance of defining and capacity to consent so that mental health research is done in accordance with the research ethics.

**Individual 3:** they are afraid of being stigmatized- we can overcome the problem by teaching mindset change to society

**Individual 4:** lack of awareness on the assessment or defining mental health statuses.

**Individual 5:**  when we go to communities, we often don’t explain in detail the intent, we assume they are always ready and willing to attend to us and answer our questions. So, we have to explain in details for their understanding and allow them to make an informed decision.

**Individual 7:** by letting people know all the criteria involved in mental health counseling

**Individual 8:** lack of knowledge so there is need for more awareness

**Individual 10:** It's challenging in a sense that there is no prioritization.

**Individual 11:** they are afraid of being labeled; we can address this issue by educating society on how to change behaviour

**Individual 12: l**imited knowledge on **mental** health research and ethics on researchers

**Individual 6 and 9 skipped the question**

**Q16 In your context, are there any other limits or barriers to the inclusion of people with lived experience in mental health research and how do you overcome these barriers?**

**Individual 2**: no there are no any other limits or barriers.

**Individual 3:** there are several barriers; Not considered as a development issue by the government and its partners. Not supported financially.

**Individual 4:** through awareness campaigns

**Individual 5:** yes, stigma. Allocate more attention and support to mental health issues.

**Individual 9:** due of the barriers is cultural values perceive one with mental health as weak. The solution is to sensitize communities that everyone can have mental problems.

Respondents 1,6, 7, 8, 10, 11, 12

**Q17 In your context, does mental health research receive sufficient resources? o Yes (1) o No (2)**

**11** individuals= No all others

**1** Individual = Yes respondent 9

**Q18 If no, please describe what resources are needed and what impact you think this has?**

**Individual 1**: Financial capacity, personnel, research capacity. This lack of inadequate of the above-mentioned resources slows down the work.

**Individual 2:** there are inadequate human resources and financial resources which create negative impacts in addressing the mental health challenges.

**Individual 3:** financial and technical support needed to ensure that these challenges are addressed.

**Individual 4:**  NGOs tend to ignore it and focus on their issues

**Individual 5:** funds to run programs that support mental health, in order to address mental health issues in our communities.

**Individual 7:** need for financial resources, need for expertise to train people who deal with mental health issues

**Individual 8:** To address these problems, financial and technical aid is needed.

**Individual 10**: Non-governmental organizations (NGOs) often disregard it in lieu of concentrating on their own issues.

**Individual 11:** money for mental health initiatives to address mental health issues in our communities.

**Individual 12:** financial means, as well as the ability to teach those who work with mental illness, are required.

Respondents - 6 and 9 did answer

**Q19 In your context, who sets the priorities for mental health research? ▢Academia (1) ▢Civil Society (2) ▢Government (3) ▢Medical Profession (4) ▢People with Lived Experience (5) ▢None of these (6)**

**Individual 1:** Academia

**Individual 2:** civil society

**Individual 3:** Academia

**Individual 4:** academia,

**Individual 5:** academia

**Individual 6:** civil society, medical profession, people with lived experience

**Individual 7:** Academia

**Individual 8:** academia,

**Individual 9:** academia

**Individual 10:** Academia

**Individual 11:** academia,

**Individual 12:** academia

**Q20 If none of these, please explain further. If more than one, how are the priorities different?**

Ignored as it was not applicable since they selected optons in question 19

**Individual 1:** The academia, medical profession provides the information through research findings. The government develops policies and approves the implementation. The civil society advocates for the implementation of such policies.

**Individual 4:** government and civil society need to work on policy implementation and the academia needs to conduct more research.

**Q21 In your context, do you think that religious, spiritual and other local belief systems have a role in mental health research? o Yes (1) o No (2)**

12 individuals= Yes

**Q22 If yes, how would you describe this role?**

**Individual 1:** These systems are influencers, therefore have a role to play in the provision of the right information to people

**Individual 2:** Realizing and appreciating that mental health challenges affect the congregants and followers of religious and spiritual groupings they have a significant role to play in creating awareness and managing mental health among followers

**Individual 4:** to enable their followers change their mindset and starting regarding mental health as a disease like any other diseases.

**Individual 5:** they influence the mental statuses

**Individual 7:** awareness of the problem to mentors

**Individual 10:** religious and local leaders are trusted by communities where they rule issues, so they can be used to counsel those with mental health issues.

**Individual 12: N/A**

Respondents 3,6, 8, 9, 11, skipped

**Q23 How do you rate the workshop you attended and what improvements would you like to see in future workshops?**

**Individual 1:** 9/10, it was very good and insightful

**Individual 2:** the workshop was very beneficial and an eye opener to me personally and the organization I represented in general

**Individual 3:** very helpful. Improvements- increase time allocation

**Individual 4:** 10/10

**Individual 5:** very good initiative will help us improve how we do things in our different organizations. Looking forward to more of these

**Individual 6:** the workshop was okay but would have appreciated if it was for a few more days not just short time we had

**Individual 7:** good- more explanation on the toolkit needed

**Individual 8:** it was an eye opener, it really assisted that we need to have eye on all research node

**Individual 9:** prepapred us to be be active from the beginning of the research till the end

**Individual 10:** needed to have more time to learn more on the research journey

**Individual 11:** there is need to increase participants working around mental health as this workshop was helpful for change in the future

**Individual 12**: workshop also should have included more concetration on emerging issues from the practice in Malawi

**Q24 What do you think are the main strengths of this toolkit?**

**Individual 1:** It was very specific and the guidelines are commonly known guidelines, just further highlighted.

**Individual 2:** The main strength of this toolkit is its focus in the 4Ps

**Individual 3:** interactive

**Individual 4:** it identified the gaps in research ethics and things that are overlooked.

**Individual 5:** interaction and sharing ideas

**Individual 6:** it provides a guide on how we can conduct research with ethics issues addressed

**Individual 7:** breakdown the different areas of consideration for ethical research

**Individuals 8-12** did not answer

**Q25What aspects do you think could benefit from further development in this toolkit?**

**Individual 2:** the aspect of translating the toolkit into local languages and print into many booklets

**Individual 3:** continue collaboration and sharing of ideas

**Individual 7:**  implementing an ethical board for research and NGOs could access the research approval

**Individual 10:** the recommendations that we made will help to make a difference in our work places

**Individuals 1, 4, 5,6,8,9,11 did not answer**

**Q26 How can this toolkit be useful in your role as a researcher? How could we improve this toolkit to make it more useful for global / cross-cultural researchers?**

**Individual 2:** This toolkit can be used as roadmap in ethics practice in my research, keeping me alert on ethics throughout the process. The toolkit needs to be available to every researcher easily

**Individual 3:** it will be used for my regular reference in our work

**Individual 7:** will not only make it use but more importantly share with colleagues

**Individual 8:** it could be useful in the things that are overlooked in 4Ps. It could be improved by involving more organizations

**Individual 10:** I will always keep in mind not to infringe ethical rights of my clients

**Individual 11:** this toolkit will enhance the acceptability of researches amongst communities as it acts as generally accepted guidelines.

**Individual 12:** more example of how it relates to everyday NGO work

**Individuals 1, 4, 5, 6,9 did not answer**

**Q27 How can this toolkit be useful for ethics committees, research support offices or agencies that fund research? How could we improve this toolkit to make it more useful for ethics committees or agencies that fund research?**

**Individual 1:** this can guide funders in incorporation of the policies that are inclusive of ethics and priories good ethical conducts rather than just focusing on what they want to get out of the research.

**Individual 2:** the toolkit will be useful to the ethics committees as it will provide them with updates on approach towards research in mental health

**Individual 3:** regard mental health as a development issue

**Individual 4:** insist on the importance of ethics on research

**Individual 5:** this toolkit can be useful if more stakeholders are involved in its development

**Individual 7:** highlighting challenges unique areas where research is done

**Individual 8**: see mental health to be a developmen issue

**Individual 10**: stress the relevance of research ethics

**Individual 11:** if more stakeholders are connected to the development of this toolkit, it will be more useful.

**Individual 12:** identifying unique research problems

Individuals 6,9, did not answer

**Q28 How can this tool be useful to NGOs doing research?**

**Individual 1:** this can help NGOs have a clear roadmap and to pay attention to ethical practice in all their researches.

**Individual 2:** the toolkit can be useful to NGOs doing research as it will provide guidelines on mental health research

**Individual 3:** make it accessible to other NGOs through the CONGOMA portal

**Individual 4:**  following it and taking into consideration the 4Ps and working on their dilemmas

**Individual 5:** help with ethical consideration

**Individual 7:** it will guide the NGO to conduct the research within acceptable guidelines

**Individual 8:** explain context and importance of research in NGO

**Individual 10:** it helps to critically think about ethics on each and every node of research

**Individual 11:** It will direct the NGO's study in accordance with accepted norms.

**Individual 12:** describes the background and value of research in a non-profit organization.

**Individuals 6,9, did not answer**

**Q29 How do you rate the template we used for the workshop? o Very good (4) o Good (5) o Poor (6) o Very Poor (7)**

**Individual 1:** Very good

**Individual 2:** very good

**Individual 3:**  very good

**Individual 4:** very good

**Individual 5:**  good

**Individual 7:** very good

**Individual 8:** good

**Individual 9:** very good

**Individual 10:**  good

**Individual 11:** very good

**Individual 12:** good
