# Supplementary material for: Exploring ethical practice in NGOS on mental health research in Malawi
Source: PLOS Glob Public Health. 2024 Apr 11;4(4):e0003001. doi: 10.1371/journal.pgph.0003001 (PMC11008845; doi:10.1371/journal.pgph.0003001)
Supplement: S3 File — (DOCX) [file pgph.0003001.s003.docx]

**Research for NGOs in Malawi**

**PROGRAM**

1. Welcome Remarks
2. Understanding of Mental Health
3. NGOs understanding of Research Ethics
4. Ethical Research Concerns for NGOs in Malawi
5. Ethical practice for NGOS in Malawi

NOTES

28th June 2021, Crossroads Hotel, Blantyre.

**Facilitators:** Action Amos and Parth Patel

The discussion that took place in the workshop regarding each item on the agenda is summarized in this report via the facilitators' handwritten notes and through participant presentations.

1. **Understanding of Mental Health**

Workshop Plenary Discussion Understanding Mental Helath

**Summary**

3 Groups presented their discussion by highlighting that Mental ill-health is understood to be “punishment from the creator”, “a curse that comes your way by chance”, “generational evil deeds”. Others related it to behavioural symptoms and other that it was a medical conditions. Others combined the at least two as they indicated it can be a combination. All 15 participants agreed that this is well known and accepted conceptualization of the large populace in the Malawi. Fig A below sums the discussion.

Fig A: Summary on understanding mental health

1. **NGOs understanding of Research Ethics**

People spoke their views in groups and one on one answering how they understood research ethics.

Participant 2: Before research get **consents**, if the participants required are under age it is very important to get consent from their parents. Participants also need to be given all the respect they need regardless of their background and everything concerning both their physical or mind outlook.

Participant 1: the research should benefit the people in their **communities respectively**. We also have to consider if the people are **participating** in the research or not. If there not participating they cannot be ethics.

Participant 3: transparency

This is very important because people have to know what they are involving themselves in. **transparency** is key

Participant 4: accountability and inclusion

We should and ought to be held accountable for the proceedings of the research.

Workshop Group Discussion Pairs.

1. Standards and principles guiding the research, ie; the rights of the participants and the researchers.
2. Research process be beneficial to the communities, ie; vulnerable groups have to be protected.
3. 3) Ethics guidelines, Getting approval from the National ethics committee.
4. 4) Getting consents from the participants.
5. Working in respect of cultural and religious beliefs.
6. Transparency, disclosure of information about the research. & Accountability to the community about the proceedings of the research.
7. Non Maleficence, do not harm.
8. Beneficence, do good.
9. consent and many others were put forward.

"As far as I can tell, it alludes to the national ethics approval committee's requirement that study protocols be followed......."

In plenary individual provided feed back

"The ethics committee developed standards and principles controlling the research; this is more to do with ethical committee approvals," Individual 3: "Coming from a background of health and human rights rights, this is a bit confusing confusing because ethics are observed when you include beneficiaries in all research stages, It demonstrates how ethical the study is." "As part of my training in clinical practice, it is the practice of not harming others while patients are in the therapeutic setting," said Individual 5 (Key Informant/Individual 6).

1. **Ethical Research Concerns for NGOs in Malawi**

Workshop Group Plenary Discussion

Key Factors affecting ethical practice displayed on charts(summarised)

- "Power Balance,"

- "Ethical Engagement,"

- "Ownership,"

- "Agenda Setting"

1. **Ethical practice for NGOS in Malawi**

Group discussions on ethical problems produced the following conclusions.

1. *Delayed approvals - Mitigation: moving forward despite denials*
2. *Insufficient support for the activity's mitigation and relocation*
3. *Money/funds to support research*
4. *Protection: Adhere to our spending plan in any case*
5. *Recognition of research results*
6. *Keep using our findings as mitigation.*
7. *Obeying regulations and directives*
8. *Prevention: Conducting follow-ups*
9. *Collaboration with other partners on how they manage research problems and challenges.*
10. *Agenda setting*

*Another group came up with the following*

- 1. *Mitigation: have a readily available research team.*
  2. *Inadequate funding - Mitigation: have strains of funding.*
  3. *Lack of expertise in research ethics- Train the team in research ethics*
  4. *Collaboration*
  5. *Agenda setting*

Summary

Key Common from two groups – agenda setting, collaboration capacity,
